# A Mixture of Tocopherol Acetate and L-Menthol Synergistically Promotes Hair Growth in C57BL/6 Mice

**DOI:** 10.3390/pharmaceutics12121234

**Published:** 2020-12-18

**Authors:** Seunghyun Ahn, Jung Yeon Lee, Sang Mi Choi, Yujeong Shin, Seyeon Park

**Affiliations:** Department of Applied Chemistry, Dongduk Women’s University, Seoul 02748, Korea; mistahn321@naver.com (S.A.); dwd924@hanmail.net (J.Y.L.); smchoi113@naver.com (S.M.C.); yujeing@naver.com (Y.S.)

**Keywords:** synergistic effect, transcriptome analysis, functional gene clustering, skin development, hair cycle-related gene

## Abstract

Oral finasteride and topical minoxidil are single components approved by the US FDA for treating hair loss. Some other compounds originating from natural products are also traditionally used for promoting hair growth. In this study, observations of treated keratinocyte cells were used to demonstrate that tocopherol acetate, L-menthol, and stevioside exert an effect on cell regeneration. Furthermore, these were topically applied to the shaved skin of C57BL/6 mice to observe their effects on hair growth. A mixture of tocopherol acetate, L-menthol, and stevioside showed the highest potential for promoting hair growth in vivo. In in vivo experiments, the mixture of tocopherol acetate, L-menthol, and stevioside was more effective than tocopherol acetate or L-menthol alone in promoting hair growth. The transcriptome analysis of skin from the dorsal side of a mouse treated with tocopherol acetate or L-menthol versus vehicle revealed key changes in keratin, keratin-associated protein, forkhead box, sonic hedgehog, fibroblast growth factor 10, desmoglein 4, deoxyribonuclease 1-like 2, and cadherin 3, known to play roles in promoting hair growth.

## 1. Introduction

The treatment of hair loss is limited due to the lack of therapies to induce and sustain remission [1]. Although there are a variety of alternative medicines for treating hair loss, only oral finasteride (Proscar^®^) and topical minoxidil (Rogaine^®^) are products approved by the US FDA for hair loss treatment. Many traditional alternative medicine remedies have been used for hair loss for centuries. However, only a few are backed by scientific evidence and the results of randomized controlled trials. In addition, there is a lack of standardization of the bioactive ingredients in alterative and traditional medicine. In particular, a few single components have been reported to be able to promote hair growth with effects on gene regulation. Some previous studies have examined the hair growth-promoting effects of tocopherol acetate, L-menthol, and stevioside [2,3,4].

It is known that alopecia is associated with oxidative stress. Tocopherol has been reported to possess high antioxidant potency. The effect of orally administered tocotrienol (a tocopherol derivative) on hair growth has been investigated in volunteers with alopecia [2], showing that volunteers in the tocotrienol supplementation group had significant increases in the numbers of hairs than those in a placebo group, most likely due to the antioxidant activity of tocotrienols that can reduce oxidative stress in the scalp [2]. However, the effects of tocopherol administered by topical application on hair growth in animal models or humans through clinical evaluation have not been reported yet. Regarding the effect of menthol on hair growth, an experimental trial using peppermint oil that included menthol, but not menthol as a single component, in C57BL/6 mice was performed [3]. The results show that a 4-week regimen of topical peppermint oil induced increases in dermal thickness, follicle number, and follicle depth. A stevia extract containing stevioside can also enhance the appearance of mammalian hair/fur when it is given as an oral nutraceutical or topical treatment [4].

In this study, the results show that, separately, tocopherol acetate, L-menthol, and stevioside promote the proliferation of HaCaT keratinocyte cells and that L-menthol promotes the proliferation of both dermal papilla cells and HaCaT keratinocytes. However, when tocopherol acetate, L-menthol, and stevioside were each applied topically to shaven skin of C57BL/6 mice, only tocopherol acetate and L-menthol alone were effective in promoting hair growth. A mixture of tocopherol acetate, L-menthol, and stevioside was more effective than tocopherol acetate or L-menthol alone in promoting hair growth in vivo. Tocopherol acetate and L-menthol upregulated genes known to promote hair growth, including keratin, keratin-associated protein, forkhead box, sonic hedgehog, fibroblast growth factor 10, desmoglein 4, deoxyribonuclease 1-like 2, lim homeobox protein, and cadherin 3.

## 2. Materials and Methods

### 2.1. Cells Culture and Proliferation Assay

Human keratinocytes (HaCaT) were purchased from the Korean Cell Line Bank (Seoul, Korea) and maintained in Dulbecco’s Modified Eagle Medium (DMEM) (Gibco, Carlsbad, CA, USA) supplemented with 10% fetal bovine serum (FBS) and penicillin–streptomycin (P/S). Primary human hair follicle dermal papilla cells (HDPs) were purchased from Cell Engineering for Origin (Seoul, Korea) and grown in human hair follicle dermal papilla cell growth medium (CEFOgro^TM^ HDP) in a humidified incubator at 37 °C with 5% CO_2_. A cell proliferation assay was performed using an MTS (3-(4,5-dimethylthiazol-2-yl)-5-(3-carboxymethoxyphenyl)-2-(4-sulfophenyl)-2H-tetrazolium, inner salt) assay kit (Promega, Madison, WI, USA). Briefly, cells were seeded onto 96-well plates at a density of 1.5 × 10^4^ cells/well and treated with tocopherol acetate, L-menthol, stevioside, and minoxidil at various concentrations (0, 0.2, 1, 5, 10, and 50 μM) for 24 and 48 h. These experiments were performed in triplicate. After incubation, 20 μL/well of MTS solution was added to each well. After incubating at 37 °C in a humidified 5% CO_2_ atmosphere for 1 h, the optical density was measured at 490 nm using an ELISA reader (Bio-Tek, Winooski, VT, USA).

### 2.2. Ethics Approval

All animal experiments were carried out with the approval of the Dongduk Women’s University Animal Ethics Committee (No. 201910-01). Five-week-old female C57BL/6 mice (18–20 g) were purchased from Daehan Bio Link Co. (Incheon, Korea). These mice were maintained with a standard laboratory diet under a controlled temperature (25 ± 3 °C) and a 12/12 h light/dark cycle for 7 days prior to experiments.

### 2.3. Measurement of Hair Growth

C57BL/6 mice were divided into six groups (five animals per group) (group A: the vehicle control; group B: minoxidil 3%; group C: tocopherol acetate; group D: stevioside; group E: L-menthol; and group F: a mixture of L-menthol, tocopherol acetate, and stevioside). The concentrations of these agents (0.5%) were determined by the half-maximal value of a standard used to prepare nonmedical cosmetic products. The hair on the dorsal portion of each animal was shaved off with a razor blade. Samples at various concentrations were topically applied to the shaved areas of mice once per day for eight weeks. The hair growth-promoting effect was evaluated based on the hair growth area and skin color, indicating telogen-to-anagen conversion [5]. Mice were sacrificed at eight weeks, and skin sections were obtained from the center of the treated areas, embedded in TRIzol reagent (Invitrogen, Carlsbad, CA, USA), and frozen for RNA extraction.

### 2.4. Expression Profile Analysis

To obtain RNAs for gene expression profiling, pieces of dermal tissues were lysed. Total RNA was isolated using an RNeasy Mini Kit (Qiagen, Hilden, Germany) following the manufacturer’s protocols. The purity and integrity of the isolated RNA were analyzed with an Agilent 2100 Bioanalyzer and an RNA 6000 Nano-Chip (Agilent Technologies, Böblingen, Germany). Total RNAs from the control (CTR) and treatment groups were used for transcriptome profiling. Libraries were constructed using a QuantSeq 3’mRNA-Seq library prep kit (Lexogen, Vienna, Austria). A NextSeq 500 (Illumina, San Diego, CA, USA) was used as the sequencing platform, and the library layout was determined using an Illumina SE75.

### 2.5. Statistical Analysis

Data are presented as mean ± SD of three or five independent experiments. In all experiments, statistically significant differences were determined using Student’s *t*-test (Sigma Plot, La Jolla, CA, USA). *p* < 0.05 was considered statistically significant.

### 2.6. Data Analysis

QuantSeq 3’mRNA-Seq was performed by eBiogen (eBiogen Inc., Seoul, Korea). The sequences were aligned using Bowtie2 [6]. Bowtie2 indices were generated from genome assembly sequences and representative transcript sequences from the genome and transcriptome, respectively. The alignment file was used to assemble transcripts, estimate their abundance, and detect differential gene expression. Differentially expressed genes were determined based on counts from unique and multiple alignments using coverage in Bedtools [7]. The read count data were processed based on a quantile normalization method using EdgeR within R using Bioconductor [8]. The gene classification was based on searches performed against the DAVID (http://david.abcc.ncifcrf.gov/) and Medline (http://www.ncbi.nlm.nih.gov/) databases.

## 3. Results

### 3.1. Effect of Tocopherol Acetate, L-Menthol, and Stevioside on Proliferation of Keratinocytes and Dermal Papilla Cells

**Figure 1:**
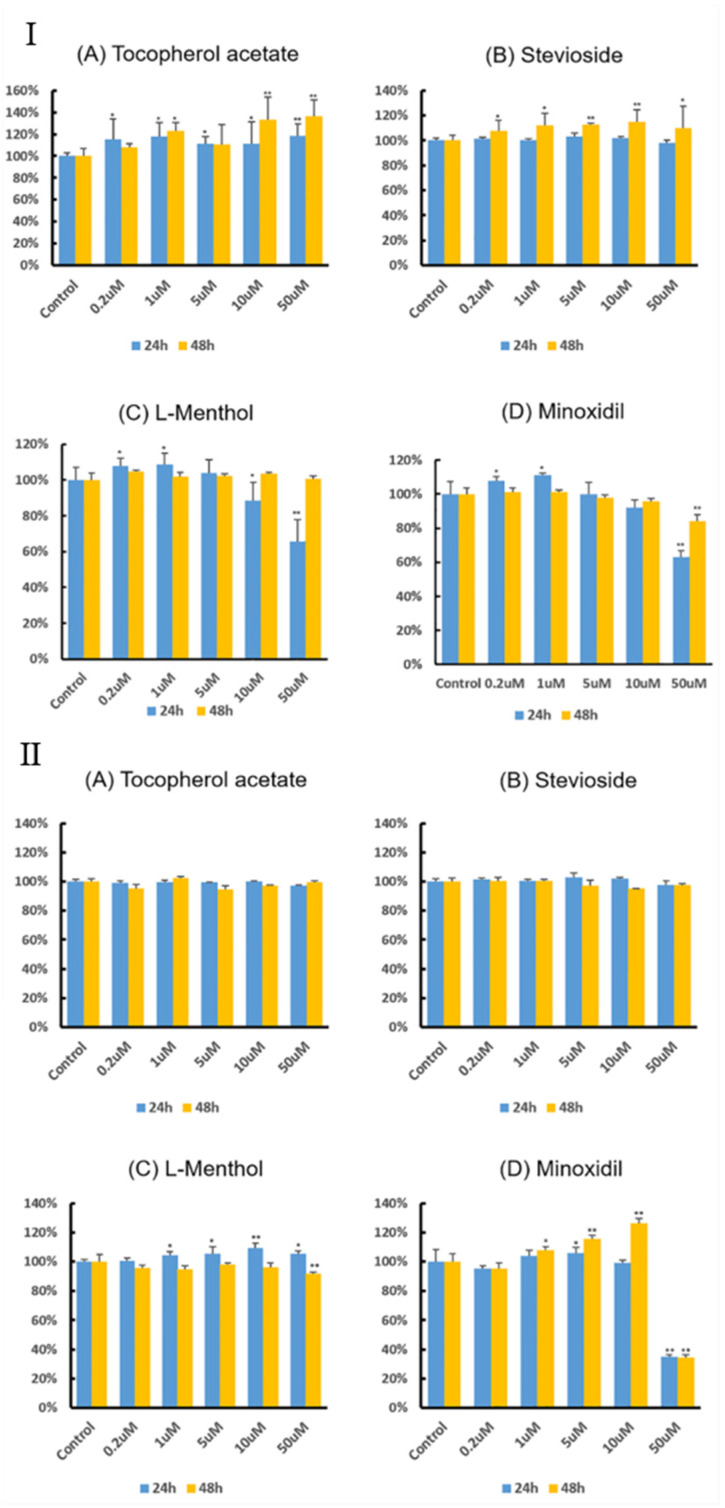
**I** Effects of (**A**) tocopherol acetate, (**B**) stevioside, (**C**) L-menthol, and (**D**) minoxidil on keratinocyte proliferation. Cells were treated with each compound at indicated concentrations for 24 and 48 h. Results are presented as average and standard deviation. Statistical analyses were performed in comparison with the vehicle-treated control group (* *p* < 0.05; ** *p* < 0.01; *n* = 6). **II** Effects of (**A**) tocopherol acetate, (**B**) stevioside, (**C**) L-menthol, and (**D**) minoxidil on human hair follicle dermal papilla (HDP) cell proliferation. Cells were treated with each compound at indicated concentrations for 24 and 48 h. Results are presented as average and standard deviation. Statistical analyses were performed in comparison with the vehicle-treated control group (* *p* < 0.05; ** *p* < 0.01; *n* = 3).

As shown in Figure 1I, treatment with L-menthol, tocopherol acetate, and stevioside alone promoted the proliferation of human keratinocytes. Tocopherol increased the proliferation of human keratinocytes in a dose- and time-dependent manner. Compared to the control (CTR), the proliferation of HaCaT cells was increased by different treatments, showing the following results: a 33% increase by 10 μM tocopherol acetate after 48 h, 15% increase by 10 μM stevioside after 48 h, and 9.5% increase by 2 μM L-menthol after 24 h. However, L-menthol was cytotoxic at concentrations higher than 10 μM. For human hair dermal papilla cells, tocopherol acetate and stevioside alone failed to significantly affect cell proliferation (Figure 1II). Treatment with L-menthol or stevioside for 48 h resulted in cytotoxicity to human hair follicle dermal papilla (HDP) cells. Stevioside was associated with 3–5% cytotoxicity in human HDP cells at 48 h. L-menthol treatment at 50 μM for 48 h resulted in cytotoxicity of 10%. Minoxidil increased the proliferation of human HDP cells in a dose- and time-dependent manner at concentrations up to 10 μM. However, 50 μM minoxidil was cytotoxic. Minoxidil at low concentrations (<10 μM) increased the proliferation of keratinocytes. However, at concentrations over 10 μM, it decreased the proliferation of keratinocytes.

### 3.2. In Vivo Evaluation of Hair Growth

**Figure 2:**
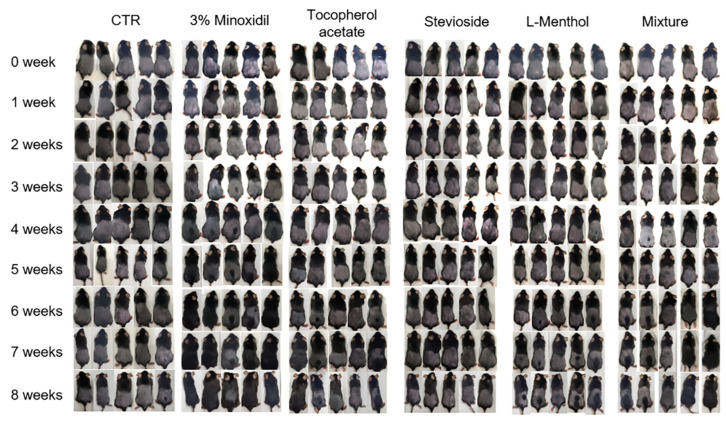
Effects of topical application of tocopherol acetate, stevioside, L-menthol, and their mixture on hair growth induction in C57BL/6 mice. Mice were divided into 6 groups: CTR (control), minoxidil, L-menthol, tocopherol acetate, stevioside, and L-menthol + tocopherol acetate + stevioside. Time scale of hair regrowth in C57BL/6 mice during 8 weeks post-shaving. The skin on the back of each mouse was photographed every week.

**Figure 3:**
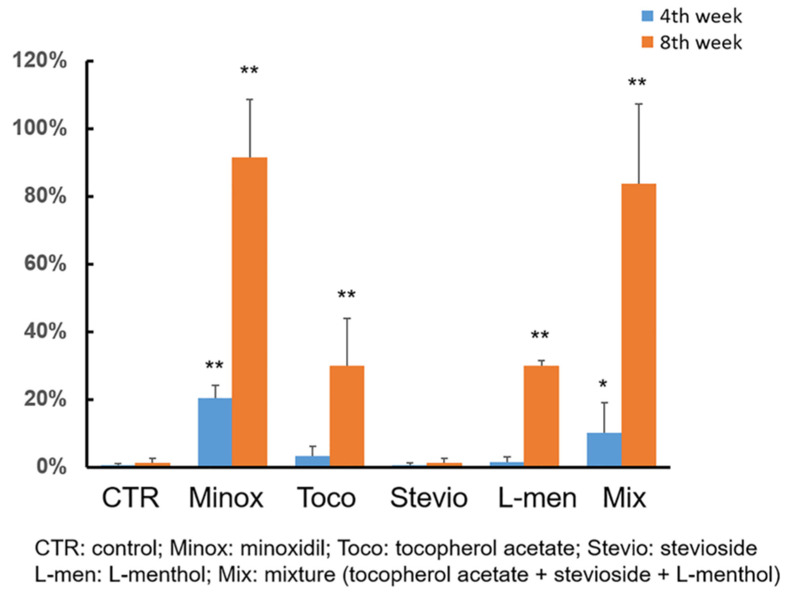
Quantitative comparison of hair growth. The hair growth area was scanned and measured with Image J. The value is represented as the percentage of the hair growth area relative to the shaved area. The average value is shown, with error bar indicating standard deviation. The calculations excluded the highest and lowest values in each group. Statistical analyses were performed in comparison with the vehicle-treated control group (* *p* < 0.05; ** *p* < 0.01; *n* = 3).

As shown in Figure 2 and Figure 3, the topical application of 0.5% tocopherol acetate or L-menthol significantly promoted hair growth in C57BL/6 mice. The group treated with a mixture of tocopherol acetate, L-menthol, and stevioside showed the highest hair growth-promoting effect. A grey spot appeared on the back of a mouse in each group. The skin first turned from pink to grey at an earlier time point in the group treated with a mixture of tocopherol acetate, L-menthol, and stevioside (13 days) compared to the vehicle control (47 days) or the 3% minoxidil (21 days) treatment group. For the quantitative comparison of hair growth, the hair growth area was scanned into a computer and the size of the area was calculated using the ImageJ program. As shown in Figure 3, treatment with tocopherol acetate or L-menthol produced a greater effect on hair growth within 4 weeks compared to the control or 3% minoxidil treatment.

Treatment with either tocopherol acetate or L-menthol resulted in a 30% increase in hair growth compared to the control. However, stevioside did not show a hair growth-stimulating effect, although it stimulated the proliferation of keratinocytes in vitro. A mixture of tocopherol acetate, L-menthol, and stevioside synergistically promoted hair growth. Their effect was greater than the summed effects of L-menthol and tocopherol acetate at 0.5% alone. The expected value of the relative hair growth area achieved by a mixture of L-menthol and tocopherol acetate could be calculated using Colby’s equation, as follows: expected value in each treatment (E) = value of L-menthol (X: 0.299) + value of tocopherol acetate (Y: 0.300) − XY/100. The achieved value of the relative hair growth area induced by treatment with the mixture of tocopherol acetate, L-menthol, and stevioside was 83.7%, which was more than the calculated expected value of 59.8%, indicating a synergistic effect of the mixture.

Grey spots appeared at 3 weeks in the 3% minoxidil group and the mixture-treated group, while grey spots appeared at 4 weeks in the L-menthol or tocopherol acetate-treated group. Of the five mice treated with the mixture of tocopherol acetate, L-menthol, and stevioside, four showed hair regrowth within five weeks. However, three and two of the five mice treated with tocopherol acetate and L-menthol, respectively, showed hair regrowth within five weeks.

### 3.3. Expression Levels of Hair Growth-Related Genes in Tissues Showing New Hair Growth

**Figure 4:**
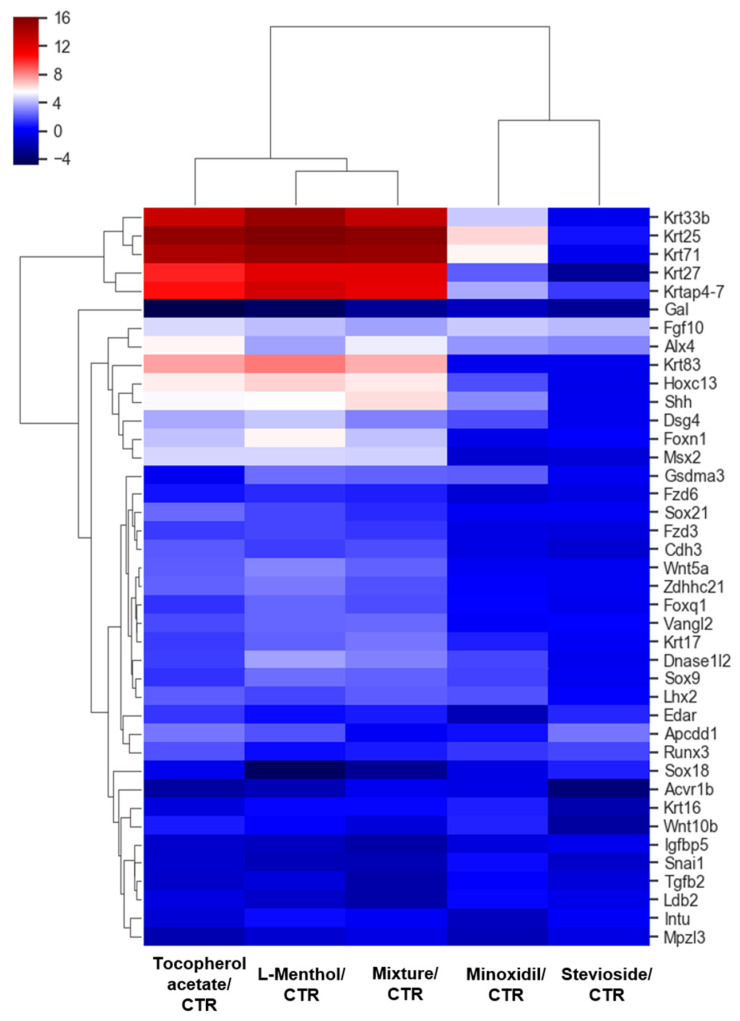
Heat map of gene expression levels for tocopherol acetate vs. CTR, L-menthol vs. CTR, L-menthol + tocopherol acetate + stevioside vs. CTR, minoxidil vs. CTR, and stevioside vs. CTR. Significant genes were defined as those with a 3-fold change, normalized data of 5.00, and *p*-value < 0.05. Hierarchical clustering was performed in the functional category of hair growth. CTR = control

Mouse back skin was used for mRNA extraction and transcriptome sequencing. We performed Quan-Seq analysis to comprehensively profile differential gene expression in mice of the vehicle group and treatment groups (3% minoxidil, tocopherol acetate, stevioside, L-menthol, and a mixture of tocopherol acetate, stevioside, and L-menthol). We identified about 400 genes that were upregulated by over 10-fold in the mixture-treated group vs. CTR. Of these ~400 genes, about 300 were commonly upregulated with at least a 2-fold change in minoxidil vs. CTR, tocopherol acetate vs. CTR, and L-menthol vs. CTR, indicating the upregulation of common sets of genes in mice showing hair regrowth after treatment with L-menthol and tocopherol acetate. The gene expression patterns in the mice showing hair regrowth differed from those seen in the stevioside treatment group (Figure 4).

The top 50 most upregulated genes in the minoxidil group were also upregulated at the highest rate in tocopherol vs. CTR and L-menthol vs. CTR (Table 1). Many of these 50 most upregulated genes in the minoxidil-, tocopherol acetate-, and L-menthol-treated groups were keratin genes or keratin-associated protein genes. We performed transcriptome annotation according to hierarchical gene ontology categories provided by the European Bioinformatics Institute. The fold-change patterns in genetic expression in tocopherol vs. CTR and L-menthol vs. CTR were very similar. According to hair regrowth annotation, about 35 genes were commonly upregulated and about 10 genes were downregulated in tocopherol vs. CTR and L-menthol vs. CTR. Besides keratin genes, *Hoxc13, Fgf10, Dnase1l2, Sox9, Lhx2, Lgr4, Runx3*, and *Sostdc1* were upregulated in mice showing hair regrowth (Table 1).

As shown in Figure 4, a gene categorization chart was constructed for significantly up- and downregulated genes (fold change: 2.00; normalized data: 4.00; *p*-value < 0.05). The list of genes was assigned to the following functional categories: cell cycle (total gene number, *n* = 1119), cell differentiation (*n* = 3697), migration (*n* = 850), extracellular matrix (*n* = 505), immune response (*n* = 1075), inflammatory response (*n* = 490), secretion (*n* = 508), hair growth (*n* = 104), positive regulation of anagen (*n* = 80), regulation of timing of anagen (*n* = 29), negative regulation of anagen (*n* = 237), and positive regulation of catagen (*n* = 347). Tocopherol acetate and L-menthol showed very similar category distribution patterns. In L-menthol-treated mice, the expression levels of 39 hair growth-related genes out of a total of 104 genes were significantly altered (33 upregulated, six downregulated), representing 37.5% functional reactivity, which was higher than the changes seen in the other gene categories. In tocopherol acetate-treated mice, the expression levels of 47 hair growth-related genes out of a total of 104 genes (36 upregulated, 11 downregulated) were significantly changed, representing 45.2% functional reactivity, which was higher than the changes seen in the other gene categories.

Tocopherol acetate and L-menthol significantly altered the expression of genes related to hair growth, the positive regulation of anagen, and the extracellular matrix at higher rates than other gene categories. Similar patterns were observed for the mixture-treated group and minoxidil-treated group. However, stevioside significantly regulated 199 immune response genes (173 upregulated and 26 downregulated), a very high rate of regulation in that category.

**Figure 5:**
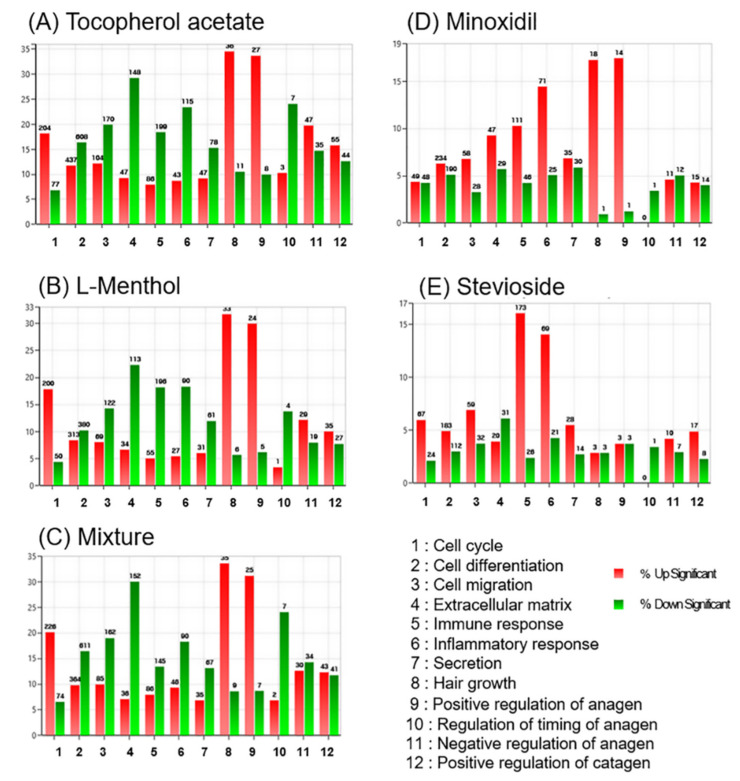
Gene category chart of (**A**) tocopherol acetate vs. CTR, (**B**) L-menthol vs. CTR, (**C**), L-menthol + tocopherol acetate + stevioside vs. CTR, (**D**) minoxidil vs. CTR and (**E**) stevioside vs. CTR. CTR = control

**Figure 6:**
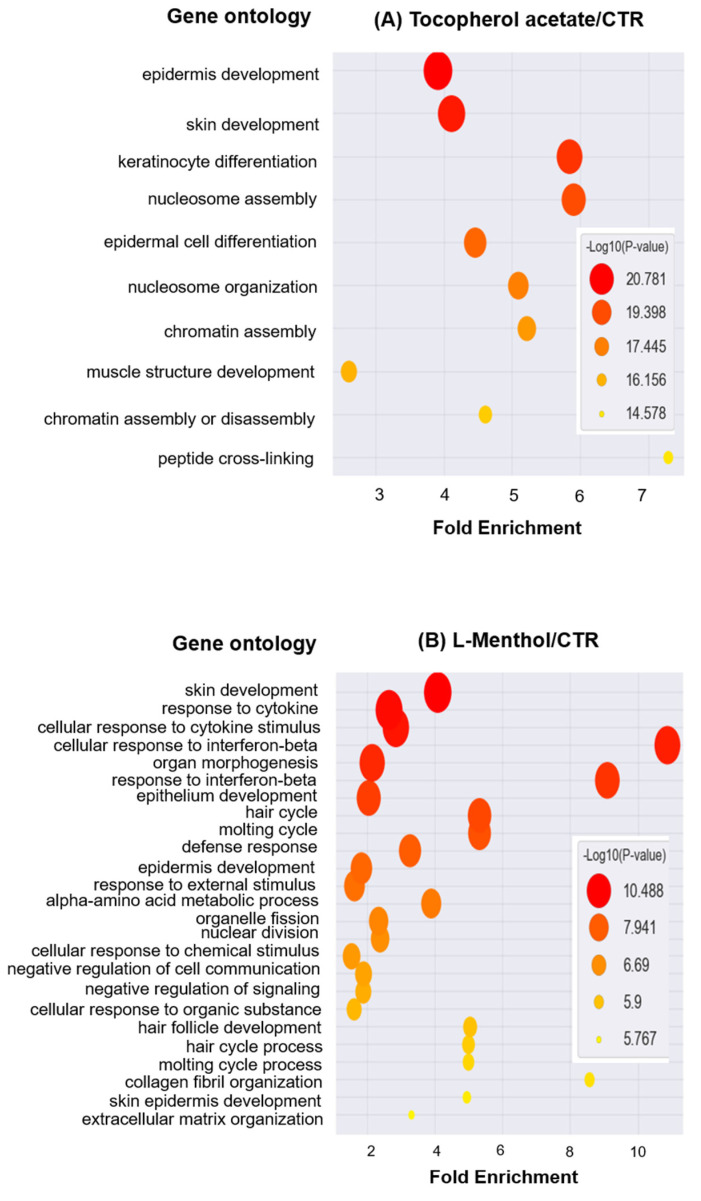
Functional annotation enrichment for tocopherol acetate (**A**) and L-menthol (**B**) treatment groups. DAVID bioinformatics resources 6.8 analysis tool was used.

The DAVID bioinformatics resources 6.8 analysis tool (NIH) was used to functionally annotate our transcriptome data. In the gene ontology biological processes generated by tocopherol acetate, epidermal development, keratinocyte differentiation, and epidermal cell differentiation were highly ranked, as shown in Figure 5. Additionally, L-menthol generated gene ontology biological processes including hair cycle, molting cycle, epidermis development, and hair follicle development (Figure 6). The list of hair cycle-related genes in the gene ontology biological processes generated by tocopherol contained *Gprc5d* (G protein-coupled receptor, family C, group 5, member D), *Sox18* (SRY (sex determining region Y)-box 18), *Sox9*, *Dnase1l2*, *Dsg4* (desmoglein 4), *Fgf10* (fibroblast growth factor 10), *Foxn1* (forkhead box N1), *Foxq1* (forkhead box Q1), *Gal* (galanin), *Hoxc13* (homeobox C13), *Krt17* (keratin 17), *Krt25* (keratin 25), *Krt27, Krt71, Krt83, Krtap21-1* (keratin associated protein 21-1), *Krtap4-16* (keratin associated protein 4-16), *Msx2* (msh homeobox 2), *Ptgs2* (prostaglandin-endoperoxide synthase 2), *Shh* (sonic hedgehog), *Vangl2* (vang-like 2 van gogh, Drosophila), and *Zdhhc21* (zinc finger, DHHC domain containing 21). The list of hair cycle-related genes in the gene ontology biological processes generated by L-menthol included *Gprc5d* (G protein-coupled receptor, family C, group 5, member D), *Sox21* (SRY (sex determining region Y)-box 21), *Alx4* (aristaless-like homeobox 4), *Fgf10* (fibroblast growth factor 10), *Foxn1* (forkhead box N1), *Gal* (galanin), *Hoxc13* (homeobox C13), *Krt25* (keratin 25), *Krt27* (keratin 27), *Krt71* (keratin 71), *Krt83* (keratin 83), *Krtap21-1* (keratin associated protein 21-1), *Krtap4-16* (keratin associated protein 4-16), *Msx2* (msh homeobox 2), *Ptgs2* (prostaglandin-endoperoxide synthase 2), *Shh* (sonic hedgehog), *Tnf* (tumor necrosis factor), and *Zdhhc21* (zinc finger and DHHC domain containing 21).

## 4. Discussion

The present study determined the hair growth-promoting efficacy of a mixture of tocopherol acetate, L-menthol, and stevioside. The effect of the mixture on mice was almost the same as that of minoxidil. In our in vivo experiment, tocopherol and L-menthol alone showed a hair regrowth effect. The skin in the vehicle control group became black in 4–5 weeks, whereas that in the tocopherol acetate or L-menthol group began to become dark in 4 weeks. The skin in the minoxidil group and the mixture group became black skin in 3 weeks, representing the fastest hair growth rate.

This is the first study investigating the hair growth-promoting effect of a mixture of tocopherol acetate and L-menthol as the active ingredients. Although tocopherol acetate, L-menthol, and stevioside alone increased the proliferation of HaCaT cells, stevioside did not show any in vivo hair growth-promoting effect. Stevioside did not promote the growth of human HDP cells either. Tocopherol acetate treatment produced an in vivo hair growth effect, although it did not promote the proliferation of human HDP cells. Minoxidil promoted the proliferation of both keratinocytes and human HDP cells at concentrations between 0.2 and 10 μM at 48 h. However, it showed cytotoxicity at 50 μM (0.001%) or higher, which was a much lower concentration than the physiological dose concentrations (3–5%).

The hair shaft is made up of epidermal keratinocytes in the hair matrix that encloses the dermal papilla and dermal papilla cells in the bulb [9]. The bulge and the dermal papilla cells contained therein play key roles in hair growth (anagen phase) and regeneration (transition from telogen phase to anagen phase) [10]. HDP cells interact with surrounding epithelial cells, resulting in hair shaft elongation by the proliferation and differentiation of the epithelium of the bulb during the anagen phase [9]. Adequate epithelial–HDP communication is important for hair follicle growth and cycling [11]. In particular, the HDP lies adjacent to keratinocytes and produces signals including insulin-like growth factor-1, hepatocyte growth factor, and vascular endothelial growth factor crucial for regulating keratinocyte proliferation and differentiation in the hair shaft [12,13]. Therefore, HDP cells can act as the “command center” for the process of hair growth [13,14]. Furthermore, the number of HDP cells is correlated with the size and shape of the hair shaft in the follicles of mice [15]. Moreover, the number of HDP cells increases during the anagen phase of the human hair cycle [16]. To evaluate the potential of a new candidate hair growth-promoting compound, researchers generally ask whether the material can promote the proliferation of keratinocytes or human HDP cells. However, our results indicate that there are some limitations of measuring the proliferation of HDP cells and keratinocytes to determine the hair growth-promoting effects of various materials.

We performed differential gene expression analysis between the control group (vehicle) and the group treated with minoxidil, tocopherol acetate, stevioside, or L-menthol. There were some similarities in terms of differentially expressed genes between the tocopherol acetate and L-menthol treatment groups, while there were rarely similarities in the genes that were differentially expressed between the stevioside group and minoxidil group or between the stevioside group and tocopherol acetate or L-menthol group. In our transcriptome-based hierarchical clustering data, the top 200 most upregulated genes in the tocopherol acetate treatment group compared to the vehicle control group were also upregulated in the L-menthol treatment group, indicating that these genes were related to hair growth. KEGG (Kyoto Encyclopedia of Genes and Genomes) pathway analysis performed using DAVID gene ontology biological processes implied that cGMP–PKG, PPAR, and cAMP/MAPK signaling pathways were involved in the tocopherol acetate treatment group. PPAR controls fatty acid metabolism. Its upregulation in the hair bulb is closely related to hair miniaturization and concurrent androgenic alopecia upregulation [17]. Furthermore, the activation of the Wnt/β-catenin and MAPK pathways in the hair follicle can mediate hair growth promotion [18]. Genes such as *FABP3*, *FABP6*, *PPARα*, and *PPARγ* known to be involved in the PPAR pathway were downregulated by tocopherol treatment. These results could be used to elucidate precisely how tocopherol acetate promotes hair growth. The KEGG pathways in the L-menthol treatment group contained PI3K–Akt, NF-kappa B, cAMP, and TGF-β signaling pathways and the ECM–receptor interaction pathway. TGF-β has been previously shown to be important for regulating the hair cycle and hair growth [19].

Many hair regrowth-related genes were upregulated in the tocopherol acetate and L-menthol treatment groups. Hoxc13 is known as a crucial regulator of the murine hair cycle [20]. The keratinocyte-specific endonuclease DNase1L2 has been previously reported to play an essential role in the removal of nuclear DNA from hair and nails [21]. DNase1L2 is an essential and specific regulator of programmed cell death in skin appendages. Its involvement in hair regrowth demonstrates that the breakdown of nuclear DNA is crucial for establishing the full mechanical stability of hair [22]. The transcription factor Sox9 is also known to be pivotal in the morphogenesis of hair follicles [23]. Trichohyalin is a large structural protein abundant in the inner root sheath of anagenic hair follicles. It could mediate keratin filamentous assembly by changing granule formation and rearranging the keratin meshwork [24]. The proteinaceous components of the hair shaft consist largely of keratin intermediate filaments, keratin-associated proteins, and V-set and immunoglobulin domain containing 8 (VSIG8), which are subjected to transglutaminase cross-linking in the hair shaft and precortex of the hair follicle bulb [25]. VSIG8 is expressed at the late anagen and early catagen stages of the hair cycle [25]. Peptidyl arginine deiminase, type I encodes a member of the peptidyl arginine deiminase family of enzymes. It catalyzes the post-translational deimination of proteins by converting arginine residues into citrullines in the presence of calcium ions [26]. Its type I enzyme is involved in the late stages of epidermal differentiation by deaminating filaggrin and keratin K1 to maintain the hydration of the stratum corneum, hence having a cutaneous barrier function [26]. This enzyme may also play a role in hair follicle formation [26].

A health food supplement containing Chinese herbal medicine has been put forth as a hair growth promoter. Its mechanism of action in improving androgenetic alopecia is potentially associated with the increased expression of EGF, FGF5, and FGF7 [27]. In the current work, when significant genes were filtered for hierarchical gene ontology categories of hair growth, regulation of catagen or anagen, and EGF/PDGF signaling, we found that tocopherol acetate and L-menthol regulated 35 and 29 hair growth-related genes, respectively. Tocopherol acetate and L-menthol also upregulated a positive anagen gene regulator, *Wnt5a*.

Although stevioside alone did not show any effect on hair growth in mice in the present study, it did regulate six hair growth-related genes and downregulated one *galanin* gene involved in positive catagen regulation. Stevioside influenced epidermal development and keratinocyte differentiation processes. However, the major gene ontology biological process affected by stevioside was the immune response-related process. Hair loss due to damage from T cells has been recently characterized as an autoimmune condition [28]. The present functional annotation study of stevioside provides an immune process-related explanation for the ambiguous role of stevioside in hair growth induced by the mixture of tocopherol acetate and L-menthol. Additional investigation needs to be carried out to determine whether this compound contributes to the promotion of hair growth. Stevioside is known to cause the inhibition of calcium influx via the blockage of calcium channels, inhibition of extracellular calcium influx, and release of prostaglandins [29,30]. In addition, stevioside exerts anti-inflammatory and immunomodulatory activities by actively attenuating the nuclear factor (NF)-kappa B pathway [31]. Stevioside also shows reactive oxygen species-scavenging ability, leading to a decrease in cellular stress [22].

The synergistic effect of tocopherol acetate, L-menthol, and stevioside on hair growth promotion should be further investigated. Notably, our synergistic effect supports the known ability of stevioside to enhance the bio-efficacy of other substances. A previous report has shown that stevioside can enhance the bioavailability of anti-tubercular, anti-leprotic, anti-cancer, antifungal, and anti-viral drugs when given at oral doses of 0.01–50 mg/kg of body weight [32]. Although the mechanism of action is not fully known, the effect of stevioside as a bioenhancer may be exerted following topical application and be relevant to hair growth promotion.

According to the functional gene clustering analysis, many gene ontology biological processes were generated by minoxidil, including ion transport, calcium ion signaling, epidermal development, keratinocyte differentiation, hair follicle development, and hair cycle and molting cycle processes. The epidermal development and keratinocyte differentiation-related list of genes affected by minoxidil treatment included *Lhx2* (LIM homeobox protein 2), *Sox9* (SRY (sex determining region Y)-box 9), cystatin E/M *(Cst6)*, deoxyribonuclease 1-like 2 *(Dnase1l2)*, fibroblast growth factor 10 *(Fgf10)*, homeobox C13 *(Hoxc13)*, keratin 10 *(Krt10)*, keratin 2 *(Krt2)*, keratin 25 *(Krt25)*, keratin 27 *(Krt27)*, keratin 36 *(Krt36)*, keratin 6A *(Krt6a)*, keratin 6B *(Krt6b)*, keratin 71 *(Krt71)*, keratin 84 *(Krt84)*, keratin associated protein 21-1 *(Krtap21-1)*, late cornified envelope 1L *(Lce1l)*, late cornified envelope 3A *(Lce3a)*, late cornified envelope 3D *(Lce3d)*, late cornified envelope 3E *(Lce3e)*, late cornified envelope 3F *(Lce3f)*, potassium large conductance calcium-activated channel, subfamily M, alpha member 1 *(Kcnma1)*, proline rich 9 *(Prr9)*, small proline-rich protein 1A *(Sprr1a)*, small proline-rich protein 1B *(Sprr1b)*, small proline-rich protein 2D *(Sprr2d)*, small proline-rich protein 2E *(Sprr2e)*, small proline-rich protein 4 *(Sprr4)*, stratifin *(Sfn)*, deoxyribonuclease 1-like 2 *(Dnase1l2)*, keratin 10 *(Krt10)*, keratin 2 *(Krt2)*, keratin 6A *(Krt6a)*, keratin 6B *(Krt6b)*, late cornified envelope 1L *(Lce1l)*, late cornified envelope 3A *(Lce3a)*, late cornified envelope 3D *(Lce3d)*, late cornified envelope 3E *(Lce3e)*, late cornified envelope 3F *(Lce3f)*, proline rich 9 *(Prr9)*, small proline-rich protein 1A *(Sprr1a)*, small proline-rich protein 1B *(Sprr1b)*, small proline-rich protein 2D *(Sprr2d)*, small proline-rich protein 2E *(Sprr2e)*, small proline-rich protein 4 *(Sprr4)*, and stratifin *(Sfn).*

Considering all the functional gene clustering results, tocopherol acetate and L-menthol shared many similar gene regulation processes with minoxidil, including epidermal development, keratinocyte differentiation, hair follicle development, and hair cycle and molting cycle processes. However, each compound affected different pathways.

## Figures and Tables

**Table 1:** Top 22 most upregulated genes in the minoxidil group were also upregulated at the highest rate in tocopherol vs. CTR and L-menthol vs. CTR. CTR = control.

ID	Gene Symbol	Minoxidil/CTR	Tocopherol Acetate/CTR	L-Menthol/CTR	Mixture/CTR
3639	*Krt25*	260.007	69,758.518	38,576.555	49,921.374
7399	*Krt71*	244.293	40,677.839	19,642.107	33,811.480
3686	*Krt33b*	188.837	33,661.314	8375.150	10,228.858
3641	*Krt27*	159.456	3959.954	1126.435	4065.116
3662	*Krtap4-7*	157.903	6715.837	1553.395	3493.634
7382	*Krt83*	112.307	326.863	187.571	159.249
7438	*Hoxc13*	105.255	96.561	63.002	65.976
11623	*Alx4*	97.411	12.862	55.517	38.361
3283	*Foxn1*	96.208	57.064	20.942	21.103
5753	*Fgf10*	87.981	19.254	28.324	13.340
15279	*Shh*	84.830	48.163	44.953	79.972
9414	*Dsg4*	81.670	22.360	14.929	8.527
8439	*Dnase1l2*	80.911	13.250	3.155	8.163
3698	*Krt17*	79.713	5.190	3.058	7.126
1074	*Vangl2*	74.617	5.593	3.689	6.089
3913	*Sox9*	68.240	6.098	2.753	5.160
11095	*Lhx2*	66.851	3.656	4.901	4.904
14151	*Zdhhc21*	65.657	7.579	5.282	4.151
20698	*Cdh3*	62.833	3.104	4.636	3.978
5140	*Foxq1*	62.137	5.564	2.666	3.879
11695	*Lgr4*	58.893	3.327	2.610	2.999
6365	*Fzd3*	58.131	3.593	2.983	2.786
